# Arterial embolization of focal nodular hyperplasia of the liver: A case report

**DOI:** 10.1016/j.ijscr.2024.109473

**Published:** 2024-03-05

**Authors:** Hiba Ben Hassine, Mohamed Ali Chaouch, Maissa Jallali, Hanen Zenati, Besma Gafsi, Faouzi Noomen

**Affiliations:** a Department of Visceral and Digestive Surgery, Monastir University Hospital, Monastir, Tunisia; b Department of Anesthesia, Monastir University Hospital, Monastir, Tunisia

**Keywords:** Focal nodular hyperplasia, Liver tumors, Aterial embolization, Conservative management, Case report

## Abstract

**Introduction and importance:**

Focal nodular hyperplasia (FNH) is a benign liver lesion that can pose diagnostic and management dilemmas, especially when distinguishing it from other hypervascular hepatic lesions. The benign nature of FNH often makes conservative management a priority; however, intervention may be necessary in symptomatic cases or when diagnostic uncertainty exists.

**Case presentation:**

A 19-year-old male presenting with abdominal pain, found to have a large 25 cm FNH lesion in the right lobe of the liver. Initial diagnosis was achieved through ultrasonography and contrast-enhanced computed tomography (CECT), with histopathological confirmation via core needle biopsy. Given the lesion's size and the patient's symptomatic presentation, we opted for arterial embolization, a less invasive surgical approach, over traditional resection methods. This technique not only led to symptom resolution but also resulted in a significant reduction in lesion size.

**Clinical discussion:**

Our approach to managing this FNH case involved a multidisciplinary team. The decision to employ arterial embolization over more invasive surgical options was based on the lesion's characteristics, the patient's age, and the potential for significant morbidity associated with traditional surgery. Arterial embolization of the FNH lesion resulted in complete resolution of symptoms and a significant reduction in lesion size, from 25 cm to 12 cm, demonstrating the effectiveness of this technique in managing large FNH lesions.

**Conclusion:**

Our findings contribute to the scientific literature by showcasing the potential of less invasive surgical techniques in the management of FNH, offering valuable insights for clinicians faced with similar diagnostic and therapeutic challenges.

## Highlights

• The case highlights how arterial embolization can be an effective treatment for FNH, leading to significant symptom resolution and size reduction of the lesion, thus contributing valuable insights into less invasive treatment options for FNH.
• The report demonstrates the importance of thorough histopathological examination and imaging techniques in achieving a correct diagnosis, particularly given FNH's often asymptomatic nature and the potential for small biopsy samples to provide limited information.
• The case presents arterial embolization as a successful treatment strategy for a patient with FNH, showcasing its effectiveness in not only alleviating symptoms but also in significantly reducing the size of the lesion.

## 1 Introduction

Focal nodular hyperplasia (FNH) is the second most common benign liver lesion, primarily affecting women of reproductive age. Characterized by hyperplastic parenchymal nodules surrounding a central scar, the etiology of FNH remains poorly understood, though it is thought to be related to localized vascular abnormalities rather than hormonal factors [1]. Unlike other hepatic tumors, FNH does not seem to have a strong association with oral contraceptive use or other known risk factors for liver disease [2]. This condition is typically asymptomatic. Previous treatment trials have largely focused on conservative management. However, intervention may be necessary for symptomatic relief, to address complications such as pain or uncertainty in diagnosis, or in the rare cases of lesion growth or hemorrhage. The risk of complications is generally low [2]. Recurrent FNH is exceptionally rare, suggesting that once appropriately managed, the long-term prognosis is excellent [1]. The aim of this case presentation, reported according to the SCARE guidelines [3], is to illustrate the diagnostic journey and therapeutic management of a patient with focal nodular hyperplasia. Through this case, we aim to highlight the challenges of diagnosing FNH, especially when biopsy samples are small, and to showcase the effectiveness of arterial embolization in the treatment of a large FNH lesion, contributing to the body of evidence that supports less invasive treatment options for this condition.

## 2 Case presentation

**Fig. 1:**
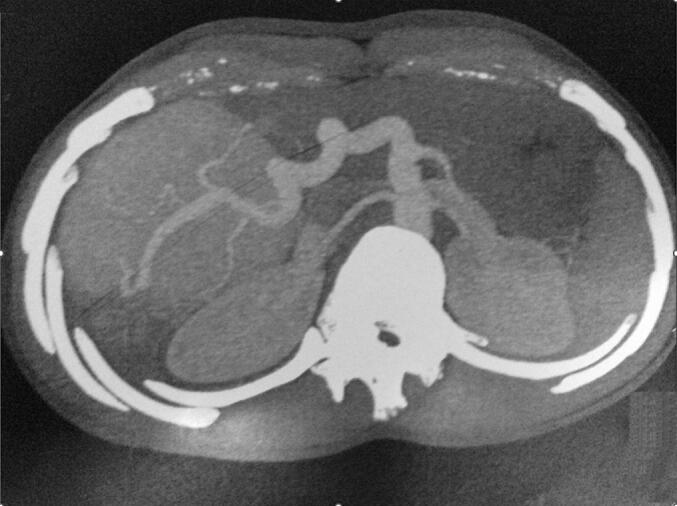
A contrast enhanced CT (CECT) scan: a large mixed density mass in the right lobe of liver with presence of a focal hypodense area in the center of the lesion.

**Fig. 2:**
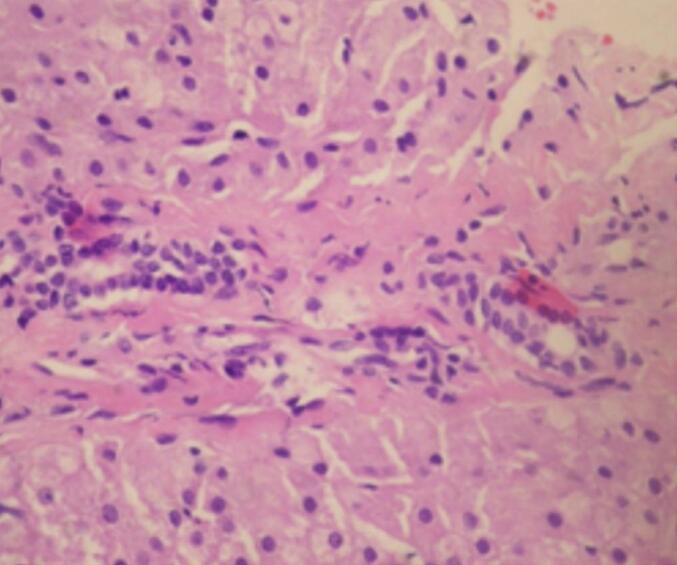
Biopsy of the liver reported the liver tissue with distorted architecture, fibrotic bands, prominent blood vessels compatible with focal nodular hyperplasia.

**Fig. 3:**
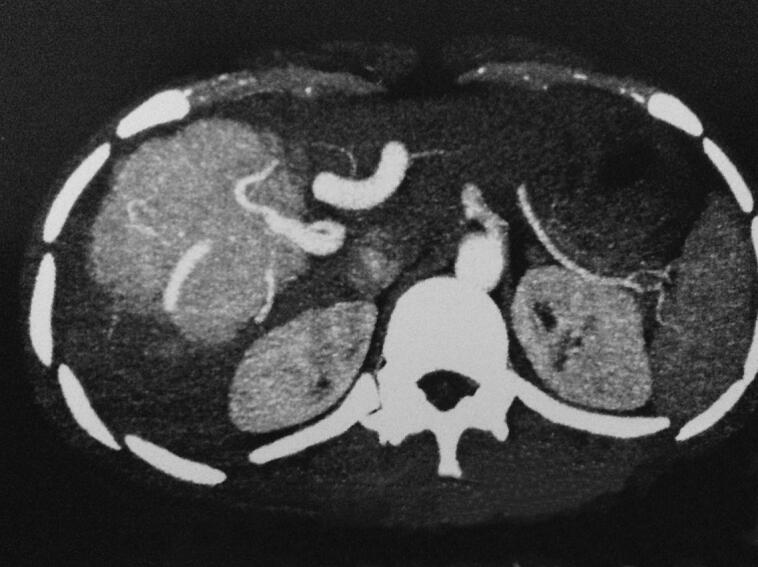
Follow-up CT-scan demonstrating significant reduction in lesion size post-arterial embolization.

A 19-year-old male with no significant past medical history or history of trauma presented for evaluation of abdominal pain persisting for one year. On physical examination, he was found to be anicteric, with a palpable mass in the right upper quadrant extending from the right subcostal region to the umbilicus and crossing the midline. Complete blood count and hemogram were within normal limits. He had previously undergone extensive investigations at another institution, where his serum alpha-fetoprotein (AFP) levels were found to be normal. Ultrasonography of the liver revealed an ill-defined, 25 cm diameter, hyperechoic, homogeneous mass lesion in the right lobe. A contrast-enhanced computed tomography (CECT) scan demonstrated a large mixed-density mass in the right lobe of the liver, featuring a focal hypodense area at the center of the lesion (Fig. 1). Microscopic examination of a core needle biopsy specimen from the liver revealed distorted architecture, fibrosis, prominent blood vessels, proliferating bile ducts, and no evidence of viral involvement or cellular atypia. These histologic findings were consistent with focal nodular hyperplasia (FNH) (Fig. 2). Given the characteristics of the FNH mass and a definitive diagnosis obtained through biopsy, arterial embolization of the lesion was performed without complications, leading to resolution of symptoms and a significant reduction in size. Follow-up imaging with a CT scan 12 months later showed a decrease in size from 25 cm to 12 cm (Fig. 3).

## 3 Discussion

To enhance the management and understanding of FNH, it is essential to consider recent advancements in diagnostic techniques, surveillance strategies, and treatment modalities, without omitting the foundational knowledge [4,5]. The management of FNH must adapt to incorporate these advancements, ensuring that patients receive the most effective and least invasive care possible. The application of cutting-edge imaging techniques, such as contrast-enhanced ultrasound (CEUS), MRI with hepatobiliary-specific contrast agents, and the use of artificial intelligence for image analysis, can improve the accuracy of FNH diagnosis [5]. These technologies can provide more detailed characterizations of liver lesions, potentially reducing the need for invasive biopsy procedures in certain cases [6]. Given the benign nature of FNH and its potential for spontaneous regression, as noted in the study monitoring FNH nodules [6], surveillance protocols can be tailored based on individual risk assessments. Factors such as the size of the lesion, growth rate, and patient symptoms should guide the frequency and type of follow-up imaging. This approach can minimize unnecessary interventions while ensuring timely detection of any significant changes in the lesion's characteristics. For symptomatic FNH or cases where intervention is deemed necessary, exploring non-invasive or minimally invasive treatment options can be beneficial. Techniques such as radiofrequency ablation (RFA) or microwave ablation (MWA) could offer alternative treatment methods for patients with specific lesion characteristics, reducing the need for open surgery and potentially decreasing recovery times [7]. The complexity of diagnosing and managing FNH underscores the importance of a multidisciplinary approach, involving hepatologists, radiologists, pathologists, and surgeons. This team collaboration facilitates comprehensive case reviews, ensuring that each patient's management plan is optimized based on the latest evidence and available technologies [8,9]. Engaging patients in the decision-making process is crucial, especially considering the benign nature of FNH and the range of management options available. Providing patients with detailed information about their condition, the potential benefits and risks of different management strategies, and the expected outcomes can help them make informed decisions about their care. Ongoing research into FNH is essential for uncovering new insights into its pathogenesis, natural history, and optimal management strategies. Collaborative efforts should aim to update clinical guidelines regularly, incorporating new evidence and reflecting the consensus among experts in the field. This will ensure that management recommendations are based on the most current understanding of FNH, offering patients the best possible outcomes. The management of FNH should evolve to integrate new diagnostic methods, refine surveillance protocols, consider non-invasive treatments, and emphasize patient-centered care. By doing so, healthcare providers can navigate the challenges associated with this condition more effectively, ensuring that patients with FNH receive the most appropriate, personalized, and minimally invasive care possible.

## 4 Conclusion

FNH is a frequently encountered benign liver lesion, usually found incidentally during routine check-ups. It typically causes no symptoms but can occasionally lead to non-specific abdominal discomfort, necessitating advanced diagnostic efforts. Diagnosing FNH is challenging and requires a multidisciplinary team, including radiologists, pathologists, and hepatobiliary surgeons, to differentiate it from other liver lesions accurately. Monitoring is preferred for asymptomatic cases, while surgery may be considered for those with symptoms or inconclusive imaging results. Ongoing research is vital to refine management guidelines and explore new treatment options for FNH.

## Patient consent

Written informed consent was obtained from the patient to publish this case report and accompanying images. On request, a copy of the written consent is available for review by the Editor-in-Chief of this journal.

## Provenance and peer review

Not commissioned, externally peer-reviewed.

## Ethical approval

Ethical approval is exempt/waived at our institution.

## Funding

No funding.

## Author contribution

All the authors participated in the manuscript and validated the final version of the manuscript.

## Guarantor

Mohamed Ali Chaouch.

## Research registration number

Not applicable.

## Conflict of interest statement

The authors declare no competing interest.
